# Impact of calcium aluminate cement with additives on dental pulp-derived cells

**DOI:** 10.1590/1678-7757-2019-0105

**Published:** 2019-11-15

**Authors:** Nadyne Saab Messias, Gabriela Grisote, Antonio Secco Martorano, Roger Rodrigo Fernandes, Ivone Regina de Oliveira, Karina Fittipaldi Bombonato-Prado, Paulo Tambasco de Oliveira, Larissa Moreira Spinola de Castro-Raucci

**Affiliations:** 1 Universidade de Ribeirão Preto, Faculdade de Odontologia, Ribeirão Preto, SP, Brasil; 2 Universidade de São Paulo, Faculdade de Odontologia de Ribeirão Preto, Ribeirão Preto, SP, Brasil; 3 Universidade do Vale do Paraíba, Instituto de Pesquisa e Desenvolvimento, São José dos Campos, SP, Brasil

**Keywords:** Odontoblasts, Root canal filling materials, Dental pulp capping, Calcium chloride, Gene expression

## Abstract

Calcium aluminate cement (CAC) has been highlighted as a promising alternative for endodontic use aiming at periapical tissue repair. However, its effects on dental pulp cells have been poorly explored. Objective: This study assessed the impact of calcium chloride (CaCl_2_) and bismuth oxide (Bi2O_3_) or zinc oxide (ZnO) additives on odontoblast cell response to CAC. Methodology: MDPC-23 cells were exposed for up to 14 d: 1) CAC with 2.8% CaCl_2_ and 25% ZnO (CACz); 2) CAC with 2.8% CaCl_2_ and 25% Bi_2_O_3_ (CACb); 3) CAC with 10% CaCl_2_ and 25% Bi_2_O_3_ (CACb+); or 4) mineral trioxide aggregate (MTA), placed on inserts. Non-exposed cultures served as control. Cell morphology, cell viability, gene expression of alkaline phosphatase (ALP), bone sialoprotein (BSP), and dentin matrix protein 1 (DMP-1), ALP activity, and extracellular matrix mineralization were evaluated. Data were compared using ANOVA (α=5%). Results: Lower cell density was detected only for MTA and CACb+ compared with Control, with areas showing reduced cell spreading. Cell viability was similar among groups at days one and three (p>0.05). CACb+ and MTA showed the lowest cell viability values at day seven (p>0.05). CACb and CACb+ promoted higher ALP and BSP expression compared with CACz (p<0.05); despite that, all cements supported ALP activity. Matrix mineralization were enhanced in CACb+ and MTA. Conclusion: In conclusion, CAC with Bi_2_O_3_, but not with ZnO, supported the expression of odontoblastic phenotype, but only the composition with 10% CaCl_2_ promoted mineralized matrix formation, rendering it suitable for dentin-pulp complex repair.

## Introduction

Endodontic therapy comprises a set of procedures aimed at the elimination of inflammatory and infectious processes and the promotion of an adequate environment for tissue repair to ensure the maintenance of the tooth in its alveolar process.^1^^,^^2^ Cement plays a fundamental role in this therapeutic approach as one of its desirable characteristics is the ability to stimulate the healing process of dental and periodontal tissues.^3^^–^^6^ Therefore, many research areas have focused on the development of and improvement in materials to achieve better therapeutic results.^7^^,^^8^

Calcium aluminate-based cement (CAC) represents a promising alternative since it exhibits favorable physicochemical characteristics such as its thermal coefficient of and chemical composition similar to the tooth and human bone^9^ – but also due to the bioactivity, i. e., its ability to form a biologically active apatite layer upon exposure to body fluids.^10^^–^^12^ This property, also observed in calcium silicate-based cements such as mineral trioxide aggregate (MTA), involves the calcium release and the pH raise in the milieu^13^^,^^14^ and is considered essential for the stimulating effect of these materials on cellular events that form mineralized tissues.^13^ In fact, exposure of osteoblastic cells to a CAC formulation developed for endodontic purposes (i.e., CACz with 2.8% CaCl_2_ and 25% ZnO) stimulated the gene expression of osteoblastic markers and the activity of alkaline phosphatase — an enzyme that contributes to matrix mineralization to values higher than those of the gold standard MTA.^15^ Evaluating the effects of different radiopacifier agents for CAC, all formulations exhibited low cytotoxicity on pulp cells, with behavior similar to MTA,^16^ although only Bi_2_O_3_ and ZrO_2_ (but not ZnO) conferred appropriate radiopacity on the CAC for its distinction from adjacent mineralized structures.^17^

Aiming to improve the biological and clinical properties of CACz, we proposed raising the CaCl_2_ content to increase calcium release and enhancing its radiopacity by replacing ZnO by Bi_2_O_3_.^17^^,^^18^ In osteoblastic cells, CAC with Bi_2_O_3_ and increased CaCl_2_ content supported cell differentiation and matrix mineralization.^18^ However, given that cell responses to a biomaterial might vary depending on the cell type and/or its differentiation stage,^19^^,^^20^ assessing the odontoblast response to the formulations of CAC proposed is much needed, aiming at defining the suitability of this material for dentin-pulp complex repair therapies. The null hypothesis tested was that the variations in the CAC formulation would not alter the odontoblastic cell response to the material.

## Methodology

### Odontoblast-like cell cultures

MDPC-23 cells were grown in T-75 flasks (Corning Inc., Corning, NY, USA) with 15 mL of expansion medium composed of Dulbecco's modified Eagle's medium (DMEM; Invitrogen/Thermo Fisher Scientific, Waltham, MA, USA), 10% fetal bovine serum (Invitrogen/Thermo Fisher Scientific, Waltham, MA, USA), 100 ug/mL streptomycin, and 100 UI/mL penicillin (Gibco/Thermo Fisher Scientific, Waltham, MA, USA). The cells were maintained in a humidified environment at 37°C with 5% CO_2_ and 95% atmospheric air. At subconfluence, the cells were removed with ethylenediaminetetraacetic acid (EDTA solution – 1 mM, Gibco/Thermo Fisher Scientific, Waltham, MA, USA) and trypsin (0.25%, Gibco/Thermo Fisher Scientific, Waltham, MA, USA), plated at 10 000 cells/well on Thermanox^®^ coverslips (Nunc, Rochester, NY, USA) in 24-well polystyrene plates (Corning Inc., Corning, NY, USA), and cultured in expansion medium supplemented with 7 mM β-glycerophosphate (Sigma-Aldrich, St. Louis, MO, USA) and 50 µg/mL ascorbic acid (Sigma-Aldrich, St. Louis, MO, USA) for 24 h before exposure to the cements. The culture medium was changed every three days (1 mL/well).

### Cement manipulation and culture exposure

The following cements were used: 1) CACz^15^^,^^18^; 2) CACb with Bi_2_O_3_ and 2.8% CaCl_2_; 3) CACb+ with Bi_2_O_3_ and 10% CaCl_2_; and 4) MTA (Angelus, Londrina, PR, Brazil). The CAC powders were mixed with sterile water (3:1, v/v). MTA was handled according to the manufacturer's instructions. Samples (cylindrically shaped, 2 mm in height and 4 mm in diameter) were prepared in silicon templates (Silatec, DMG Chemisch-Pharmazeutische Fabrik, Hamburg, Germany) under sterile conditions for 4 h. Then CAC or MTA samples were transferred on polycarbonate inserts (pore size 3 µm, Transwell^®^, Corning Inc, Corning, NY, USA) for cell exposure. Non-exposed cultures served as control.

### Cell morphology

Cultures were fixed on days one and three with 4% paraformaldehyde in 0.1 M sodium phosphate buffer (PB; pH 7.2) for 10 min at room temperature (∼25°C; RT), washed in PB (3x), and treated with 0.5% Triton X-100 in PB for 10 min for cell permeabilization. Cells were incubated with Alexa Fluor™ 488 (green fluorescence)-conjugated phalloidin (1:200, Molecular Probes/Invitrogen, Eugene, OR, USA) for 60 min at RT for actin cytoskeleton labeling. Cells were washed with deionized water and cell nuclei marked with 300 nM 4’,6-diamidino-2-phenylindole, dihydrochloride (DAPI, Molecular Probes, Eugene, OR, USA) for 5 min. The Thermanox^®^ coverslips were mounted in Prolong Antifade reagent (Molecular Probes, Eugene, OR, USA) and the cells were observed under epifluorescence using an AxioImager M2 Zeiss light microscope (Carl Zeiss Inc., Oberkochen, Germany) outfitted with an AxioCam MRM digital camera (Carl Zeiss Inc., Oberkochen, Germany).

### Cell viability

Cell viability/proliferation was evaluated at days one, three, and seven by the mitochondrial tetrazolium test (MTT; Sigma-Aldrich, St. Louis, MO, USA).^15^ Briefly, MTT solution (5 mg/mL) was added to the culture medium at 10%, and the cells were maintained in a humidified environment at 37°C with 5% CO_2_ and 95% atmospheric air for 4 h. The solution was removed, and acid isopropanol (0.04 M HCl in 2-propanol; Sigma-Aldrich, St. Louis, MO, USA) was added (1 mL/well). The plates were shaken for 5 min. Absorbance was detected at 570 nm (μQuant™; BioTek, Winooski, VT, USA).

### Gene expression

At day seven, the culture medium was removed from the wells, and TRIzol LS reagent (Invitrogen/Thermo Fisher Scientific, Waltham, MA, USA) was added by pipetting at 25°C under agitation for 5 min. Total RNA extraction was performed with the SV Total RNA Isolation System kit (Promega, Madison, WI, USA) as indicated by the manufacturer. Then total RNA was quantified (260, 280, 230, and 320 nm) on a spectrophotometer (GE Healthcare Life Sciences, Marlborough, MA, USA). The cDNA was made from 1 μg of total RNA. The procedure was performed in a Mastercycler Gradient Cycler (Eppendorf, Hamburg, Germany) using the GoScript™ Reverse Transcriptase (Promega, Madison, WI, USA) kit following the manufacturer's instructions. For the real-time PCR reaction, the GoTaq^®^ qPCR Master Mix reagent (Promega, Madison, WI, USA) and the StepOnePlus™ Real-Time PCR System (Applied Biosystems/Thermo Fisher Scientific, Foster City, CA, USA) were used. The sequences of the primers used in this study were: 1) alkaline phosphatase (ALP) forward ATC TTT GGT CTG GCT CCC ATG, reverse TTT CCC GTT CAC CGT CCA C; 2) dentin matrix protein 1 (DMP-1) forward GGA GCA AGG TGA CAG CGA GT, reverse GAG ACT GGA GGC CTT CCT GG; 3) bone sialoprotein (BSP) forward GAC TGC TTT AAT CTT GCT CTG CAT, reverse GTA GCG TGG CCG GTA CTT AAA; and 4) β-actin (ACT) forward GCT GAC AGG ATG CAG AAG GA, reverse TGG ACA GTG AGG CCA GGA TA. Reactions were performed in triplicate with a final volume of 10 µL containing 12.5 ng of cDNA. Amplification reactions were performed under the following cycling conditions: 2 min at 95°C followed by 40 cycles of 15 s at 95°C and 1 min at 60°C. The β-actin gene was used as an endogenous control. The comparative 2^–∆∆Ct^ method was used to compare the gene expression levels of cultures from the different experimental groups. The results were expressed as gene expression relative to the control group.

### ALP activity

ALP activity was evaluated *in situ* at days 7 and 10 using fast red TR dye. After culture medium was removed, the wells were washed with PBS and incubated in a humidified environment at 37°C with 5% CO_2_ and 95% atmospheric air for 30 min with 1 mL of Tris-buffered solution (120 mM, pH 8.4, Sigma-Aldrich, St. Louis, MO, USA) containing naphthol-AS-MX-phosphate (0.9 mM; Sigma-Aldrich, St. Louis, MO, USA) fast red TR (1.8 mM; Sigma-Aldrich, St. Louis, MO, USA), and dimethylformamide (1:9; Sigma-Aldrich, St. Louis, MO, USA). ALP activity was assessed from the macroscopic images of the cultures.

### Mineralized bone-like nodule formation

Matrix mineralization was evaluated by alizarin red staining (ARS) at day 14. The cultures were fixed in 70% ethanol for 60 min at 4°C, washed in PB, and stained for 15 min with 2% ARS (Sigma-Aldrich, St. Louis, MO, USA) at pH 4.2 at RT. Macroscopic images were obtained for qualitative evaluation of matrix mineralization.

### Statistical analysis

Data (n=4) were carried out using ANOVA followed by the Student-Newman-Keuls *post hoc* test when appropriate (α=5%).

## Results

### Cytotoxicity of CAC formulations on odontoblast-like cells

**Figure 1:**
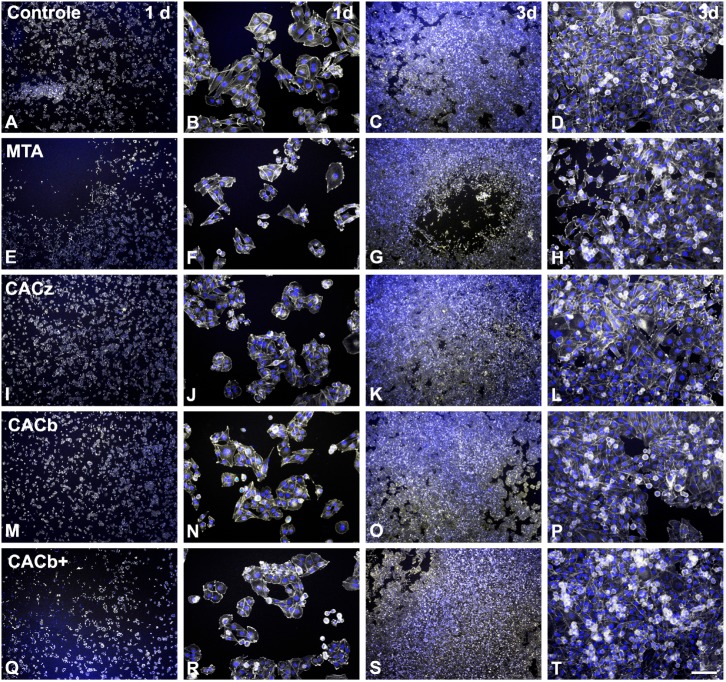
Morphological aspects of MDPC-23 odontoblast-like cell cultures exposed or not (Control, A-D) to MTA (E-H), CACz (I-L), CACb (M-P), or CACb+ (Q-T) cements at days 1 (A, B, E, F, I, J, M, N, Q, R) and 3 (C, D, G, H, K, L, O, P, S, T). Green fluorescence (grayscale mode) indicates the actin cytoskeleton, and blue fluorescence, cell nuclei. Scale bar= 800 μm for A, C, E, G, I, K, M, O, Q, and S; 100 μm for B, D, F, H, J, L, N, P, R, and T

Epifluorescence revealed lower cell density in cultures of MDPC-23 cells exposed to the endodontic cements compared with control cultures at day one, particularly in MTA (Figure 1, E) and CACb+ (Figure 1, Q). In both cases, the cultures exhibited a central region devoid of cells in areas closer to the cement samples. Also, cells exposed to MTA and CACb+ showed reduced cell spreading compared with control cells (Figure 1: compare B with F and R). At day three, only cultures exposed to MTA exhibited a central area devoid of cells (Figure 1, G), while the other cultures presented morphological aspects similar to those of the control cultures.

Cell viability increased over time for all groups, with 1 < 3 < 7 days (two-way ANOVA, p<0.001, Table 1). The comparisons between groups indicated statistical similarity for cell viability values obtained at days 1 and 3 (p>0.05, Table 1) and lower cell viability for MTA and CACb+ groups compared with the other groups at day 7 (p<0.05, Table 1).

**Table 1:** Mean ± standard deviation of cell viability at days 1, 3, and 7 in odontoblast-like cell cultures exposed or not (Control) to mineral trioxide aggregate (MTA) and calcium aluminate cement (CAC) formulations

	Cell viability		
	Day 1	Day 3	Day 7
Control	0.16±0.02^Aa^	0.56±0.02^Ab^	1,11±0.09^Ac^
MTA	0.14±0.02^Aa^	0.61±0.06^Ab^	0.82±0.11^Bc^
CACz	0.16±0.02^Aa^	0.58±0.04^Ab^	1.00±0.08^Ac^
CACb	0.17±0.03^Aa^	0.56±0.03^Ab^	1.08±0.12^Ac^
CACb+	0.18±0.03^Aa^	0.66±0.05^Ab^	0.91±0.07Bc

Different upper-case letters indicate statistical significance between lines (p<0.05), while different lower-case letters, statistical difference between columns (p<0.05)

### Effects of CAC formulations on odontoblast phenotype acquisition

ALP gene expression was higher for CACb+ compared with MTA, CACz, and control (one-way ANOVA, p<0.05, Table 2), but not compared with CACb (p>0.05, Table 2). The DMP-1 gene expression was higher for MTA and CACb+ compared with CACb, control, and CACz (p<0.05, Table 2). CACb+ showed the highest BSP gene expression levels, followed by CACb (p<0.05), whereas MTA, CACz, and control exhibited similar BSP expression levels (p>0.05, Table 2).

**Table 2:** Mean ± standard deviation of gene expression of alkaline phosphatase (ALP), bone sialoprotein (BSP) and dentin matrix protein 1 (DMP-1) at day 7 in odontoblast-like cell cultures exposed or not (Control) to mineral trioxide aggregate (MTA) and calcium aluminate cement (CAC) formulations

	Gene expression		
	ALP	BSP	DMP-1
Control	1.02±0.26^A^	1.08±0.42^A^	1.00±0.13^A^
MTA	2.38±0.51^AB^	0.69±0.30^A^	2.46±0.42^B^
CACz	1.79±0.70^A^	3.67±3.79^A^	0.15±0.06^C^
CACb	3.72±0.89^BC^	16.87±8.20^B^	0.69±0.02^AC^
CACb+	5.08±0.26^C^	30.19±3.87^C^	1.89±0.18^B^

Different upper-case letters indicate statistical significance between lines (p<0.05)

**Figure 2:**
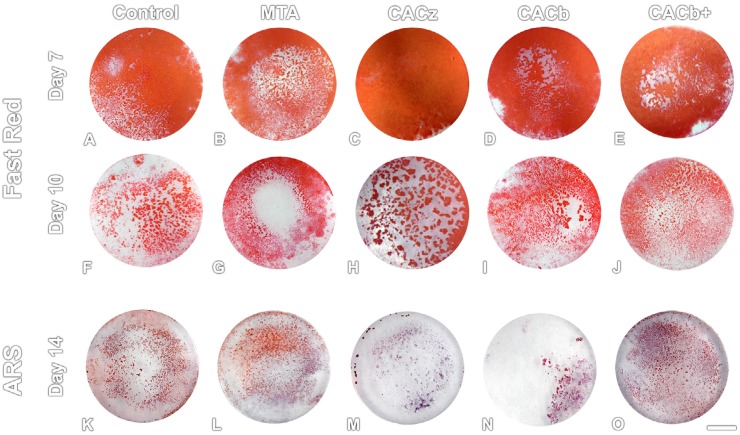
Macroscopic aspects of fast red labeling for in situ ALP activity (A-J) at days 7 (A-E) and 10 (F-J) or of alizarin red labeling for calcium deposits at day 14 (K-O) in MDPC-23 odontoblast-like cell cultures exposed or not (Control, A, F, K) to MTA (B, G, L), CACz (C, H, M), CACb (D, I, N), or CACb+ (E, J, O). Scale bar= 3 mm

Qualitative evaluation by fast red labeling revealed that all cultures exhibited ALP activity *in situ* at days 7 and 10, particularly at day 7 (Figure 2). At day 7, CACz and Control presented a larger positive area for ALP activity (Figure 2). Cultures exposed to the other cements exhibited a central area devoid of fast red labeling in areas closer to the cement samples (Figure 2). At 10 days, this unmarked central area for ALP activity was still evident for MTA and CACb, whereas for control, CACz, and CACb+, the fast red-positive areas were more homogeneously distributed by the substrate (Figure 2).

At the end of 14 days, calcium deposits marked by alizarin red were observed for all groups and were more intense in the control, MTA, and CACb+ compared with the CACz and CACb (Figure 2).

## Discussion

This study evaluated the effects of three CAC formulations developed as an alternative to MTA on dental pulp cell growth and differentiation. The null hypothesis was rejected, since the additives used in the CAC formulation altered the odontoblastic cell response to the material. We verified that replacing zinc oxide by bismuth oxide in the CAC formulations promoted gene expression of odontoblast cell markers. However, only the association of bismuth oxide with a higher CaCl_2_ content in CAC stimulated mineralized matrix formation.

Aiming to improve CAC formulations, modifications of its original additives were proposed:^11^ 1) substitution of the zinc oxide (ZnO) radiopacifying agent with bismuth oxide (Bi_2_O_3_); and 2) increase in the CaCl_2_ content to 10%, aiming for higher calcium release,^18^ which is considered to be accountable for the biological properties of MTA.^21^

By evaluating the impact of the different CAC formulations on cell growth, we found, in general, the cements were not cytotoxic and promoted viability values similar to those of MTA. Furthermore, we found replacing the radiopacifying agent by bismuth did not affect the cell viability or proliferation of the cultures even after 7 d. These findings corroborate those reported by other authors,^16^ with similar cell viability values observed after exposure of odontoblast cells to CAC with different radiopacifiers such as zinc oxides, bismuth, and zirconia. In addition, this study showed cell viability is maintained throughout the proliferative period of the culture until the seventh day. A previous study conducted by our research group showed different results in osteoblastic cultures, in which the incorporation of bismuth oxide increased the CAC cytotoxicity, promoting a significant reduction in cell viability and proliferation. These differences are likely due to the differential response between osteoblasts and odontoblasts to zinc and bismuth. In fact, in pulp-derived cells, bismuth oxide has been stimulated the expression of heme oxygenase-1,^22^ which exhibits antiapoptotic effects^23^ and stimulates cell growth,^24^ while in osteoblasts, bismuth has been related to the reduction in biocompatibility of MTA by inhibiting cell proliferation, osteoblastic differentiation, and extracellular matrix mineralization.^25^^,^^26^

The association of bismuth oxide with a higher CaCl_2_ content in CAC promoted a significant reduction in cellular viability and spreading, with results similar to those observed for MTA. These findings corroborate the results of previous investigation conducted by our research group for osteoblastic cultures.^18^ In both cases, the increased release of calcium and hydroxyl ions promoted by the cements^12^ may be involved both in the reduction in cell spreading and in the occurrence of cell-free areas near the cement, which were reflected in a lower absolute cell viability value. In fact, *in vivo*, the high pH of calcium hydroxide-based materials causes a superficial injury in the pulp tissue, promoting coagulation necrosis and tissue disorganization in the adjacent pulp tissue.^27^^,^^28^ The migration of pulp stem cells to the necrotic area can be observed only after resolution of inflammation, followed by their differentiation into odontoblast-like cells, and the deposition of a dentin-like tissue at the end of 4 weeks.^27^^–^^30^ Considering these observations, we also evaluated the effect of these preparations on the development of odontoblastic phenotype by the analysis of the gene expression of odontoblast-cell markers, ALP activity, and matrix mineralization.

The alkaline phosphatase enzyme is present in the early stages of differentiation of mineralized matrix-producing cells (osteoblasts and odontoblasts) and is responsible for the release of inorganic phosphate to form hydroxyapatite during the biomineralization process.^31^ Furthermore, acidic proteins such as bone sialoprotein and dentin matrix protein 1 are known to play an essential role in matrix mineralization through the regulation of hydroxyapatite crystal size and morphology.^32^ In this study, cultures exposed to the preparations of CAC with bismuth oxide, and particularly CACb+ (with higher calcium chloride content), exhibited the most significant levels of odontoblast cell markers, whereas CAC with zinc oxide inhibited their expression. These results may be related to the differential response of pulp-derived cells to bismuth and zinc oxide, since the exposure of cells derived from pulp to zinc oxide has been reduced expression levels of genes related to odontoblastic differentiation, including ALP.^33^

Fast red labeling revealed that cultures exposed to all of the cements showed ALP activity, particularly at seven days. As mentioned previously, ALP activity plays a key role in the eventual extracellular matrix mineralization process.^31^ The results of this qualitative analysis verified that minimal fast red labeling occurred in the central areas of the culture where the cells grew in close contact with the cement samples, especially in the MTA group. These findings are likely related to the higher concentration of ionic dissolution products of the cements in these regions, which contribute to lower cell density; this phenomenon has also been described elsewhere.^15^^,^^18^ However, in the peripheral areas of the substrate, extensive fast red-positive areas were observed in cultures exposed to CAC formulations, revealing that they promote the expression of the odontoblastic phenotype. Corroborating these findings, the presence of deposits of the mineralized matrix in all experimental groups was verified after 14 d, although the lack of calcium deposits in the central areas of the cement groups persisted.

When examining the cement groups, we verified that the substitution of zinc oxide with bismuth oxide as radiopacifier agent in CAC improved cell differentiation at gene expression level, without interfering in its potential for promoting the formation of the mineralized matrix. In contrast, increasing calcium chloride content in the CAC significantly stimulated the extracellular matrix mineralization process. The matrix mineralization process involves the deposition of calcium phosphate crystals in a fibrous extracellular matrix.^34^ Calcium ions are recognized for their ability to regulate physiological cellular processes. For instance, calcium released from materials used as pulp capping participates in calcium carbonate formation, stimulating the expression of genes related to odontoblastic differentiation and the biomineralization process.^35^^,^^36^ Given that the inclusion of bismuth oxide in CAC reduces calcium release compared with the original formulation with zinc oxide,^18^ this association with higher calcium chloride content confers superior properties on the CAC cement, also allowing for its use in regenerative procedures of the dentin-pulp complex.

## Conclusion

In conclusion, the addition of bismuth oxide to CAC associated with a higher concentration of calcium chloride enhanced odontoblast gene expression and function, offering a promising alternative to MTA for dentin-pulp complex regeneration. Nonetheless, *in vivo* studies are necessary to confirm the beneficial effects of CAC on dentin bridge formation and tissue repair.
